# Supplementary material for: Understanding the roots: Local stakeholders’ insights on the causes and challenges in combating child marriage in mountainous Karnali, Nepal
Source: PLOS Glob Public Health. 2025 Mar 18;5(3):e0004323. doi: 10.1371/journal.pgph.0004323 (PMC11918358; doi:10.1371/journal.pgph.0004323)
Supplement: S1 Text — (DOCX) [file pgph.0004323.s001.docx]

S1 Text: FGD and KII topic guide

Topic Guide

- What do you understand by child marriage?
- How common is child marriage in your community?
- What do you think are the consequences of child marriage?
- What are the causes of child marriage in your community?
- When thinking about child marriage, what do you see as factors in your community that help prevent it?

Further Questions

- At what age do girls/women usually get married in your community? At what age do boys/men usually get married?
- What are your community’s beliefs about early marriage?
- Why do girls and boys marry early in this community?
- How does early marriage affect women’s health, education, or economic status? What about the lives of their children?
- What supports, programs or schemes are available from the local government?
- Are local community groups, youth clubs, FCHVs, mothers’ groups, and NGOs active in preventing child marriage? If so, how do they contribute to its prevention?
