# Supplementary material for: Understanding the roots: Local stakeholders’ insights on the causes and challenges in combating child marriage in mountainous Karnali, Nepal
Source: PLOS Glob Public Health. 2025 Mar 18;5(3):e0004323. doi: 10.1371/journal.pgph.0004323 (PMC11918358; doi:10.1371/journal.pgph.0004323)
Supplement: S1 Table — (DOCX) [file pgph.0004323.s003.docx]

S1 Table: Consolidated criteria for reporting qualitative studies (COREQ): 32-item checklist

| **Item No** | | **Guide Questions/Description** | **Response** | |  |
| --- | --- | --- | --- | --- | --- |
| **Domain 1: Research team and reflexivity** | | | | |  |
| **Personal Characteristics** | | | | |  |
| 1. Interviewer/ facilitator | | Which author/s conducted the interview or focus group? | PH (co-author) and three other local data collection team members, acknowledged in the manuscript, conducted the interviews and focus groups. These local team members, native to Karnali province, were trained in qualitative data collection methods specific to this study. | |  |
| 2. Credentials | | What were the researcher’s credentials? E.g., PhD, MD | Four researchers collected data: Padma Hitan has a bachelor’s in public health; two researchers were health assistants, and one was an auxiliary nursing midwife. | |  |
| 3. Occupation | | What was their occupation at the time of the study? | PH was the Project Officer for the "Improving Reproductive Health and Preventing Child Marriage in Nepal and Vietnam" project, of which this study was a component. The other three research assistants served as team leaders within the same project, contributing to various activities, including data collection for this baseline study. | |  |
| 4. Gender | | Was the researcher male or female? | Two female and two male researchers | |  |
| 5. Experience and training | | What experience or training did the researcher have? | PH holds a bachelor’s degree in public health and has prior experience with the Nepal Health Research Council, where she contributed to various health research projects. Additionally, NR, who has significant experience in qualitative research, conducted an intensive three-day online training in Nepali. This training covered qualitative research methods, including techniques for key informant interviews (KIIs) and focus group discussions (FGDs), followed by a pilot test in a community outside the study area. The team also participated in a refresher course before field deployment to ensure preparedness. | |  |
| **Relationship with participants** | | | |  |  |
| 6. Relationship established | | Was a relationship established prior to study commencement? | A relationship with the participants was established prior to the study through engagement with key community stakeholders, including the Khadachakra municipality, school staff, mothers’ group facilitators and members, and youth clubs. These stakeholders were aware of and supported the project, helping to facilitate access to participants. | |  |
| 7. Participant knowledge of the interviewer | | What did the participants know about the researcher? e.g. personal goals, reasons for doing the research? | Details are included in the methodology. The research participants included beneficiaries and stakeholders of the "Improving Reproductive Health and Preventing Child Marriage in Nepal and Vietnam" project in Kalikot district. During the project’s inception meeting, many stakeholders became familiar with the project’s objectives and the data collection team’s role in conducting baseline assessments. | |  |
| 8. Interviewer characteristics | | What characteristics were reported about the interviewer/facilitator? e.g. Bias, assumptions, reasons and interests in the research topic | Details are included in the methodology. | |  |
| **Domain 2: study design** | | | |  |  |
| **Theoretical framework** | | | |  |  |
| 9. Methodological orientation and Theory | What methodological orientation was stated to underpin the study? e.g. grounded theory, discourse analysis, ethnography, phenomenology, content analysis | Grounded theory | |  |  |
| **Participant selection** | | | |  |  |
| 10. Sampling | How were participants selected? e.g., purposive, convenience, consecutive, snowball | Purposive | |  |  |
| 11. Method of approach | How were participants approached? e.g., face-to-face, telephone, mail, email | Participants were contacted either in person or by phone, depending on what was most convenient for them. | |  |  |
| 12. Sample size | How many participants were in the study? | 121 | |  |  |
| 13. Non-participation Setting | How many people refused to participate or dropped out? Reasons? | None | |  |  |
| 14. Setting of data collection | Where was the data collected? e.g., home, clinic, workplace | FGDs were conducted at schools and local gathering places, such as mothers' group meetings. KIIs were carried out at workplaces or other locations convenient for the interviewees. | |  |  |
| 15. Presence of nonparticipants | Was anyone else present besides the participants and researchers? | None. | |  |  |
| 16. Description of sample | What are the important characteristics of the sample? e.g. demographic data, date | Demographic characteristics and composition of group discussion members are presented in the supplementary files. | |  |  |
| **Data collection** | | | |  | No |
| 17. Interview guide | Were questions, prompts, and guides provided by the authors? Was it pilot tested? | Yes and yes | |  |  |
| 18. Repeat interviews | Were repeat interviews carried out? If yes, how many? | None | |  |  |
| 19. Audio/visual recording | Did the research use audio or visual recording to collect the data? | Yes | |  |  |
| 20. Field notes | Were field notes made during and/or after the interview or focus group? | Yes | |  |  |
| 21. Duration | What was the duration of the interviews or focus group? | Average interview time and FGD time were 45 minutes and 69 minutes, respectively. | |  |  |
| 22. Data saturation | Was data saturation discussed? | Yes | |  |  |
| 23. Transcripts returned | Were transcripts returned to participants for comment and/or correction? | They were not; the reason is presented under data analysis. | |  |  |
| **Domain 3: analysis and findings** | | | |  |  |
| **Data analysis** | | | |  |  |
| 24. Number of data coders | How many data coders coded the data? | Two | |  |  |
| 25. Description of the coding tree | Did the authors provide a description of the coding tree? | N/A | |  |  |
| 26. Derivation of themes | Were themes identified in advance or derived from the data? | Derived from data | |  |  |
| 27. Software | What software, if applicable, was used to manage the data? | None | |  |  |
| 28. Participant checking | Did participants provide feedback on the findings? | Yes | |  |  |
| **Reporting** | | | |  |  |
| 29. Quotations presented | Were participant quotations presented to illustrate the themes/findings? Was each quotation identified? e.g., participant number | Yes | |  |  |
| 30. Data and findings consistent | Was there consistency between the data presented and the findings? | TBD | |  |  |
| 31. Clarity of major themes | Were major themes clearly presented in the findings? | Yes | |  |  |
| 32. Clarity of minor themes | Is there a description of diverse cases or a discussion of minor themes? | Yes | |  |  |
