# Supplementary material for: Understanding the roots: Local stakeholders’ insights on the causes and challenges in combating child marriage in mountainous Karnali, Nepal
Source: PLOS Glob Public Health. 2025 Mar 18;5(3):e0004323. doi: 10.1371/journal.pgph.0004323 (PMC11918358; doi:10.1371/journal.pgph.0004323)
Supplement: S2 Table — (DOCX) [file pgph.0004323.s004.docx]

S2 Table

**General characteristics of FGD participants**

| **Group** | **Number of participants** | **Age Range** | **Gender** | **Location**  **(Ward Number)** |
| --- | --- | --- | --- | --- |
| 1 | 11 | 20-30 | Female | 11 |
| 2 | 9 | 30-50 | Female | 5,6,7 |
| 3 | 11 | 25-52 | Mixed-Male, Female | 5 |
| 4 | 10 | 13-17 | Female | 5 |
| 5 | 13 | 18-35 | Female | 11 |
| 6 | 12 | 13-17 | Male | 8 |
| 7 | 12 | 18-45 | Male | 2 |
| 8 | 12 | 18-55 | Male | 10 |
| 9 | 12 | 29-45 | Mixed-Male, Female | 1 |
| 10 | 9 | 17-55 | Mixed-Male, Female | 9 |
| 11 | 10 | 18-55 | Mixed-Male, Female | 3 |
| Total | 121 | - | - | - |

**General characteristics of KII participants**

| **Characteristic** | **N (%)** |
| --- | --- |
| **Sex** |  |
| Male | 8 (62) |
| Female | 5 (38) |
| **Age** |  |
| 26-35 | 5 (38) |
| 36-45 | 1 (8) |
| 46-55 | 7 (54) |
| **Occupation** |  |
| Government employees | 8 (62) |
| Elected members of the municipality | 1 (8) |
| Club leaders | 2 (15) |
| Journalists | 2 (15) |
| **Years of experience working in the municipality** |  |
| Less than 1 years | 1 (8) |
| 1-5 years | 3 (23) |
| More than 5 years | 9 (69) |
| **Total** | 13 (100) |
