# Supplementary material for: Understanding the roots: Local stakeholders’ insights on the causes and challenges in combating child marriage in mountainous Karnali, Nepal
Source: PLOS Glob Public Health. 2025 Mar 18;5(3):e0004323. doi: 10.1371/journal.pgph.0004323 (PMC11918358; doi:10.1371/journal.pgph.0004323)
Supplement: S3 Table — (DOCX) [file pgph.0004323.s005.docx]

S3 Table: Themes and codes (sub-themes)

| **Themes** | **Codes (Sub-themes)** |
| --- | --- |
| **Consequences of child marriage** |  |
| Maternal and child health | Maternal:  Teenage pregnancies  Unsafe abortion  Stillbirth  Malnutrition  Physical and mental health distress  Child:  Low birth weight  Malnutrition  Developmental disorders |
| Education and employment prospects for young parents | Compromised further education  Lack of parenting skills  Limited employment prospects  Poor economic status |
| Marriage and gender-related issues | High divorce rates  Polygamy  SGBV |
| **Causes of child marriage** |  |
| Individual factors | Youth-driven marriage  Poor decision making |
| Interpersonal factors | Family economic situation  Family traditions and perspectives  Knowledge and parenting competence  Digital connections |
| Community factors | Tradition and modernity synergy  Living conditions  Poor prospect |
| Policy factors | Legal safeguard for all  Policy implementation  Equity in accessing information and services  Competing priorities  Sustainability of initiatives |
