# Supplementary material for: Understanding the roots: Local stakeholders’ insights on the causes and challenges in combating child marriage in mountainous Karnali, Nepal
Source: PLOS Glob Public Health. 2025 Mar 18;5(3):e0004323. doi: 10.1371/journal.pgph.0004323 (PMC11918358; doi:10.1371/journal.pgph.0004323)
Supplement: Attachment — Submitted filename: Response to reviewers.docx [file pgph.0004323.s007.docx]

Response to reviewers

Responses to the general comments:

1. A complete copy of PLOS’ questionnaire on inclusivity in global research is included as a supplementary information.
2. A detailed Financial Disclosure statement is attached (Financial disclosure statement_PGPH-D-24-00451).
3. Data availability. We recognize the importance of data sharing in promoting research transparency and reproducibility. However, the data for this study includes audio recordings and transcripts with identifiable personal information, and given the distinct nature of the community, anonymizing the data to protect confidentiality is challenging. Direct sharing of raw data may compromise participant privacy, contrary to the commitments made under our ethical approvals. To address this, we propose an alternative: researchers who require data access can contact the corresponding author. We are open to collaborating with the Nepal Research Ethics Board to explore secure and ethical data-sharing mechanisms on a case-by-case basis, should access be essential for further research. We would be pleased to discuss this approach with the editor to ensure it meets the journal’s standards.
4. We would like to confirm that we drew the image/figure by hand.
5. As we have not yet received any comments on the discussion section, we have not made any updates to it at this stage. However, we welcome any feedback to strengthen and improve this part of the manuscript.

Responses to the specific reviewers’ comments:

Thank you for your time to review the paper and for providing insightful feedback. We have addressed the comments and updated the manuscript to address them. Detailed responses are provided in the table below.

| **Comment** | **Response** |
| --- | --- |
| Reviewer #1 |  |
| Adding more information on what has been learned from previous attempts to reduce child marriage in the Introduction would also set up more suggestions for the Discussion. | The introduction was updated to address the comment. |
| As a minor point, in the Methods it mentions "Additionally, formal written assent was obtained from the parents/guardians of participants under 18 years old." should be "formal written consent was obtained from parents/guardians and assent was obtained from children". | Changes were made to address the comment. |
| Reviewer #2 |  |
| Context is very important for reporting qualitative results and the authors need to provide it e.g. life expectancy of birth is now highest in the Karnali region so the statement like "... the province lags behind national averages on various educational and development indicators ..." must be critically reviewed. | Introduction section was updated to address this comment. |
| Line 59-60: Even though Nepal government has signed many international declarations to eliminate child marriage, its root cause at the local level (context) must be done competently. You must present them clearly in your findings. | Our findings address the local root causes of child marriage by examining how factors at various levels of the socioecological model influence policy implementation and program effectiveness in this community. We also present these factors in our discussion to highlight how local context and multi-level determinants impact efforts to reduce child marriage.  If the reviewer has specific suggestions or concerns about additional details, we would welcome further clarification to ensure our findings address this aspect comprehensively. |
| Line 74-77: Since this study is done in one of the municipality of Kalikot district, its findings are not transferable, one of the key domain of trustworthiness, even to other districts of Karnali so the title must be changed accordingly. | We agree that this study was conducted in a single municipality. We ensured this is detailed in the methods section to clarify the specific study scope. We provided detailed descriptions of the study setting, participants, and context, enabling others to assess the transferability of our findings to similar settings.  We would also like to highlight that it is common practice in research titles to reference the broader region or province to provide readers with a general geographic context, especially in areas with cultural and socio-political commonalities. Including “Karnali” in the title reflects this context and aligns with conventions for geographic reporting in public health research.  We hope this explanation addresses your concern and ensures clarity for readers while preserving regional relevance in the title. |
| Line 87-89: The deductive coding and its use in the analysis does not support the grounded theory. | The initial organization of data by topic guide questions was for practical purposes to ensure that both authors had easy access to the data in Nepali and English. However, the analysis itself was inductive: we generated themes directly from the raw data through an open-ended, exploratory approach without applying pre-existing codes. We have updated the data analysis section in the manuscript for clarification. |
| Line 90-94: Provide the topic guide as supplement material of the manuscript for review | We have included the topic guide as supplementary material. As noted in the manuscript, the data for this paper comes from a broader baseline assessment focused on improving reproductive health and reducing child marriage. The included topic guide specifically covers questions related to child marriage, aligning with the scope of this paper. |
| Line 95-101: Provide proof of credibility as the qualitative research must be an experienced one to conduct such trainings | The research was led by BA, a PhD with extensive qualitative research experience in different countries, including LMICs, and NR, a PhD student and native Nepali with over five years of experience in sexual and reproductive health (SRH) project implementation and evaluation in Nepal. Together, they developed the research process and topic guide in collaboration with INF field staff and the INF monitoring and evaluation department.  The data collection team consisted of native Karnali residents with health backgrounds who were well-acquainted with the local language, culture, and context. NR conducted an intensive three-day online training, during COVID-19 pandemic, covering qualitative research methods, including key informant interviews (KIIs) and focus group discussion (FGD) techniques, followed by a pilot test in a non-research community. The team also completed a refresher course before field deployment.  The depth and relevance of the data gathered indicate that the training effectively equipped the local research team with the necessary skills to conduct the study. |
| Line 116-128: Explain why the data matrix code was created if this research is using a grounded theory approach. If some key issues are addressed in this paper then drop in from the description too so that it will not be considered as the possible salami slicing. | We acknowledge that our explanation of the data curation process was not clear. The data matrix was not created for deductive coding but was used to organize the data by the questions in the topic guide to facilitate accessibility for both authors during analysis. This step was purely for the practical purposes of managing qualitative data in both Nepali and English. The analysis itself was carried out inductively, generating themes directly from the raw data. We have clarified this distinction in the revised manuscript.  Regarding the second comment, if we understand correctly, the reviewer refers to L123-124 (original manuscript) “*as well as perceived enablers and promising practices that are not presented in this paper.”* We took it out for clarity. |
| Line 116-128: Provide samples of transcripts of IDI and FGD with codes in as the supplement of the manuscript. Also describe how the validity of the transcripts were ensured. | To ensure transcript validity, a native Nepali-speaking author reviewed each audio recording while cross-referencing it with the written transcript. This careful process ensured accuracy and fidelity to participants' original responses.  Regarding coding, we would like to clarify that the Nepali transcripts themselves were not directly coded. Instead, transcript segments relevant to research objectives were organized by topic guide questions in both Nepali and English and compiled in a spreadsheet. This approach allowed us to isolate meaningful portions, omit off-topic discussions, and work efficiently from the spreadsheet. Codes were then applied within this organized framework, ensuring a structured and contextually relevant analysis.  If required, we are open to sharing anonymized transcript samples and their associated codes as supplementary material. |
| Line 116-128: Since thematic analysis was done, describe which thematic analysis method (e.g. Braun and Clarke?) was used and what lens (gender?) was used as well. | We did not specifically use a formalized method such as Braun and Clarke’s approach for thematic analysis. Instead, we applied a flexible, generic approach to thematic analysis where themes were generated inductively from the raw data. Our goal was to allow themes to emerge naturally without imposing predefined structures.  Regarding the lens used, we did not analyze the data through a particular thematic lens. However, gender-related themes, when relevant, were discussed as they emerged organically from the participants' responses during the focus group discussions and key informant interviews. |
| Line 116-128: This section requires detail description of the rigor of qualitative data analysis based on the Trustworthiness (credibility, dependability, confirmability and transferability), of the process as well as findings. It must include description of reflexivity as well as bracketing, if any, used during the data collection and analysis phases. | We referred to some aspects of trustworthiness in the data analysis section. To address the comment regarding Trustworthiness, the manuscript was updated. |
| Result section: Provide a table with description of IDI and FDG participants. Move the "Perceived causes of child marriage" theme in the beginning of the result section to provide the context. Explain why the socio ecological model was used only for this theme. | A table was added with a description of IDI and FGD participants  The FGD was structured using the topic guide, beginning with how stakeholders define child marriage, followed by their perceptions of its significance and consequences, before discussing its causes. We presented the data in the same order, as we believe this sequence offers a logical progression and maintains the flow of the conversation. Starting with the causes, without first establishing how child marriage is understood and its perceived impacts, might disrupt the narrative we aimed to build.  Regarding the use of the socio-ecological model, we applied it specifically to the causes of child marriage because it effectively organizes and categorizes the contributing factors at multiple levels—individual, interpersonal, community, and policy. This helps highlight the interconnectedness of these factors and the complexity of addressing child marriage. For the themes related to the definition, significance, and consequences of child marriage, the socio-ecological model did not seem as fitting, as these discussions were more straightforward and focused on understanding the phenomenon rather than analyzing the multi-layered contributing factors. |
| Result section: Explain why the institutional and policy level factors of the socio ecological factors are missing here. Provide key verbatim in the text as well. Table can be provided as an annex, do not report both in this section. | Initially, we grouped the institutional and policy levels under the societal domain. However, upon reflecting on your comment and reviewing the manuscript, we recognized the need to separate these components and incorporate more available data. We have updated the section to present the findings under distinct levels of the socio-ecological model.  Regarding the presentation of verbatim quotes, we prefer to organize them in a table within the main text rather than moving them to an annex. This approach allows all quotes related to specific themes to be viewed together in one place, providing a cohesive and comprehensive understanding of the data. By presenting the quotes in a table, readers can easily see how they align with the analysis without disrupting the flow of the narrative. However, as you suggest, we are open to including some key verbatim quotes directly in the text for added emphasis, if necessary. |
| Result section: Provide a table with themes with the associated codes along with the measure of interceder reliability with justification. | Table of themes and codes is included as a supplementary file.  In our study, the data were curated by a Nepali researcher who organized the transcripts and created a data matrix as described. The second author (BA, non-Nepali) conducted the thematic analysis. After the initial analysis by BA, both authors reviewed the themes and codes together, drawing on their combined knowledge of the local context and the data. The themes and codes were shared and discussed with the local coordinators to ensure that the themes accurately reflected the perspectives of the participants and minimized any potential bias in interpretation.  Although we did not formally measure intercoder reliability, this joint review of the themes functioned as a rigorous quality control mechanism. The involvement of both a Nepali researcher and a non-Nepali researcher with a strong background in qualitative health research added depth to our understanding of the data and strengthened the credibility of our analysis. |
| Result section: Use "Consolidated criteria for reporting qualitative research (COREQ): a 32-item checklist for interviews and focus groups" to self-assess this manuscript. Describe it in the method section and also provide it as annex (supplement material) | The COREQ checklist was completed and included as a supplementary document. |
