# Supplementary material for: Understanding the roots: Local stakeholders’ insights on the causes and challenges in combating child marriage in mountainous Karnali, Nepal
Source: PLOS Glob Public Health. 2025 Mar 18;5(3):e0004323. doi: 10.1371/journal.pgph.0004323 (PMC11918358; doi:10.1371/journal.pgph.0004323)
Supplement: Attachment — Submitted filename: Responses to reviewers.docx [file pgph.0004323.s008.docx]

Response to reviewers

Thank you for your time to review the paper and for providing constructive feedback. We have addressed the comments and updated the manuscript to address them. Detailed responses are provided in the table below.

| **Comment** | **Response** |
| --- | --- |
| Reviewer #1 |  |
| No further comments except that "Verbal consent" should be changed to "Verbal assent" for adolescents. | Thank you. The manuscript was updated to reflect this comment. |
| Reviewer #2 |  |
| The absence of inter-coder reliability is still an issue here to address the dependability of this work. | Thanks for the comment. We recognize that the description of the data analysis was unclear, so we have provided a more detailed explanation below, which is now reflected in the manuscript.  In our study, all audio files and verbatim transcripts were in Nepali, and we did not have the financial resources to translate the full transcripts into English. To facilitate analysis, a Nepali researcher (NR) created a structured data sheet. This sheet included the facilitators’ questions in the first column, followed by columns for each focus group discussion (FGD) and key informant interview (KII), where translated excerpts of responses were recorded. NR also added a separate column where he identified and recorded emerging themes/codes as they appeared in the data.  At this stage, the second author (BA, non-Nepali) independently reviewed the data sheet (without the identified codes by NR) and conducted an inductive thematic analysis. Following this, both authors collaboratively reviewed and refined the themes and codes, drawing on their combined knowledge of the local context and data. Additionally, the themes and codes were shared and discussed with the local coordinator (PH) to ensure they accurately reflected participants’ perspectives and to minimize potential bias in interpretation.  We did not formally calculate inter-coder reliability, as BA did not have access to the full Nepali transcripts and conducted the analysis using the curated data sheet. However, there were no significant disagreements between the authors regarding theme identification. |
| If a new inductive thematic analysis framework must be presented in the manuscript. | Thank you for your comment regarding the thematic analysis method used in our study. In our earlier response to your comment (“Line 116-128: Since thematic analysis was done, describe which thematic analysis method (e.g., Braun and Clarke?) was used”), we stated, “We did not specifically use a formalized method such as Braun and Clarke’s approach for thematic analysis. Instead, we applied a flexible, generic approach to thematic analysis where themes were generated inductively from the raw data. Our goal was to allow themes to emerge naturally without imposing predefined structures.”  We would like to clarify that, although we did not strictly follow Braun and Clarke’s framework, we did incorporate several key phases from their method.  As noted earlier, the first step in Braun and Clarke's method, familiarizing oneself with the data, was primarily undertaken by the Nepali investigator (NR), as they had access to the Nepali transcripts. In contrast, BA did not have direct access to the raw data (the Nepali transcripts) and worked with the prepared data sheet created by NR. Further, NR initially generated codes from the Nepali transcripts, while BA used the data sheet (which had been curated by NR) to generate further codes. However, this difference in approach did not result in discrepancies between the codes identified by the two authors.  The rest of the phases, including searching for themes, reviewing and refining themes, and naming and defining themes, were completed as suggested.  Therefore, we did not fully adhere to the framework. We have updated the manuscript to clarify this point and provide a more accurate description of our data analysis approach. |
| It is still not clear how the transcribed Nepali transcripts were translated and how its validity was ensured. | As noted in the manuscript, the audio files were transcribed verbatim in Nepali, not English. NR reviewed samples of the audio files and compared them with the transcriptions to ensure accuracy. Please let us know if further clarification is needed. |
| I would like you to declare the work done by all the authors so that the correct authorship can be awarded based on ICJME criteria. | **Below are the details of each author’s contribution:**   - **Substantial contributions to the conception or design of the work; or the acquisition, analysis, or interpretation of data for the work:**   - NR contributed to the conceptualization, design, data analysis, and interpretation.   - BA contributed to the conceptualization, design, data analysis, and interpretation.   - PH contributed to the design, data acquisition, and data interpretation.   - SF contributed to the conceptualization and design of the work. - **Drafting the work or reviewing it critically for important intellectual content:** All authors participated in drafting the work and reviewing it critically. - **Final approval of the version to be published:** All authors approved the final version of the manuscript. - **Agreement to be accountable for all aspects of the work in ensuring that questions related to the accuracy or integrity of any part of the work are appropriately investigated and resolved:** All authors agree to be accountable for the integrity and accuracy of the work. |
